# The tragedy of the emeritus and the fates of anatomical collections: Alfred Benninghoff’s memoir of Ferdinand Count Spee

**DOI:** 10.1017/bjt.2019.3

**Published:** 2019-09-13

**Authors:** Nick Hopwood

**Affiliations:** Department of History and Philosophy of Science, University of Cambridge, Free School Lane, Cambridge, CB2 3RH, UK

## Abstract

Retirement can be a significant period in modern academic careers, and emeritus professors have shaped the fates of collections in departments and disciplines. This is evidenced by reconstructing the meanings of Alfred Benninghoff’s remarkable memoir of Ferdinand Count Spee, sometime director of the anatomical institute in the University of Kiel. Thematizing the ‘tragedy’ of the emeritus, Benninghoff’s 1944 article recalls his predecessor’s possessive interactions with his collections as these approached assorted endings. With nostalgia and humour, it places the old aristocrat physically, intellectually and emotionally in a building that bombing would soon destroy. Benninghoff’s Spee retained control over the microscope slides with which he engaged colleagues in conversations about research in embryology and physiological anatomy. He lost authority over the teaching charts and wet preparations, but still said a long farewell to these things; he tried, like a conductor alone after a concert, to recapture an experience he had once shared. The elegy is interpreted as apologetic about anatomy under National Socialism, and as offering a model of collegiality. It illustrates how collections have mediated relations between scientific generations at the end of a career.

Alfred Benninghoff’s long-forgotten reminiscences of Ferdinand Count Spee, his predecessor as director of the anatomical institute in the University of Kiel, are no standard obituary. Instead of the usual biography, Benninghoff focused on the tragic role that Spee played between his retirement in 1923 and death in 1937: he embodied the emeritus professor as a type.^1^ Unconventional in the rich German culture of deference towards senior academics, the artfully constructed text highlights the end of Spee’s life with his collections: how, through his research materials, mostly microscope slides, he tried to recruit others to his interests; and how, on regular visits to the teaching charts and wet preparations, he said a long goodbye. With pathos and wit the article places a retiree and his collections in a transgenerational community and in the physical, intellectual and emotional spaces of the building they shared.^2^

Histories of *homo academicus* having neglected the last phase of a university career, accounts of the fates of collections tend to figure retirement, like death, as a moment of resilience, appropriation or disposal.^3^ Benninghoff’s unusual memoir invites reflection on a process. Drenched in nostalgia, it mainly tells of decline and loss. Yet it also testifies that an emeritus, relieved of official duties but still to some extent active in research, could keep more subtle powers. This retired collector maintained a prominent presence in an institution while his collections lingered, were restructured and came to various ends.

Published in the journal of the Anatomische Gesellschaft (Anatomical Society) in September 1944, Benninghoff’s essay is political in several ways. Benninghoff harked back to the German Empire while expressing ambivalence about modernization. He promoted his own functional approach within a Kiel tradition. More disturbingly, the piece took a stance towards National Socialism through its very silence about it. Written in the dying years of the Nazi regime, and published after Allied bombing had destroyed the collections, this elegy for a more innocent time says nothing about the persecution of some anatomists or the judicial murders in which others were complicit.^4^ It did teach considerate treatment of colleagues. The *éloge* makes vivid how retired professors’ collections have connected, and sometimes divided, the generations.

## Emeritus

The first paragraph of Benninghoff’s memoir reproduced the terse biographical entry for Spee in the chronicle of the University of Kiel; the second explained that Benninghoff would not expand on this. Rather, he would present the research as Spee had conveyed it to him, and the count’s personality as he had experienced it himself, in order to narrate not the ‘personal fate’ of one emeritus, but the exemplary ‘tragedy’ of ‘a type’ (p. 331). Addressing readers familiar with the context, Benninghoff did little explicit framing, though he provided plenty of cues. To locate the essay, and so to establish a safe distance from which to appreciate the writerly craft, I should set more of a scene before turning the page.

Emeritus seems to have been a new status for Spee’s generation. Imperial German professors, if they did not die in office, had stopped only when incapacitated or after celebrating an impressive birthday. Illness forced his own predecessor, Walther Flemming, to resign in 1902, aged fifty-nine.^5^ Professors otherwise carried on, because they identified with their calling and wished to keep income from lecture fees (in addition to their full salaries). To create opportunities for young talent, the democratic governments of the Weimar Republic mandated age limits, while recognizing emeritus status as more than simple retirement. Prussia, which had annexed Kiel as part of Schleswig-Holstein in 1867, enacted a restriction of sixty-eight in 1920.^6^

Benninghoff took up the issue in reporting Spee’s ‘smiling’ reply when asked why he had switched from the Kiel physiological institute, where he had written a dissertation with Victor Hensen, to Flemming’s anatomical one in 1887: the physiologists lived too long. Yet having acknowledged the reason for a limit, Benninghoff gave a standard counterargument, that Hensen had ‘reached a biblical age, without any waning of his energy’ (p. 338). When Flemming became ill, Spee won his wager; he first deputized and was then appointed full professor of anatomy and institute director. This post he occupied until he retired, with effect from 30 September 1923, and stayed on as emeritus in an institute run for a few years by Wilhelm von Möllendorff and then by Benninghoff himself.^7^

Benninghoff had studied medicine in Heidelberg, where the comparative anatomist Ernst Göppert was his mentor. After serving as a batallion physician in the First World War, Benninghoff followed Göppert to Marburg as second prosector. In 1924 Möllendorff, who had also written a dissertation under Göppert, called Benninghoff as first prosector to Kiel. He was soon promoted to associate professor and, when Möllendorff left in 1927, to full professor and director. Thanks to the Weimar law, he was only thirty-seven.^8^ In Kiel, he encountered Spee as a retiree, most of whose publications were already two to four decades old, who nevertheless participated in institute life.

Attitudes towards former directors ranged from the veneration that Spee expressed for Hensen and Flemming, and Benninghoff for Spee, through the exasperation that can also be read between some lines of Benninghoff’s memoir, to outright rejection.^9^ Many a conflict had centred on the rearrangement of collections or the occupancy of a room. In the bitterest then-recent dispute, Ludwig Plate had banished the zoologist Ernst Haeckel from his institute and museum and accused him of stealing books.^10^ By contrast, obituaries describe Möllendorff and Benninghoff as emollient paternalists. Benninghoff, who ‘felt himself always as father of his institute and his students’, fostered ‘intensive collaboration’ and socializing within a ‘family’ working on his research programme.^11^ This skilled and fortunate mediator between the anatomical generations had his ‘whole institute’, wife Anne-Marie, and Göppert all contribute to the textbook that Benninghoff regarded as his life’s work.^12^ Like Möllendorff, he expected an emeritus to have a room, to be included in the family and to restrain the urge to interfere.^13^ The retiree would treat his research materials as private property, while the new director took charge of the institute collections, which provided demonstration objects for teaching.

## Relations between generations

The presence of an emeritus added depth to relations between the generations, and Spee cut a distinctive figure as he shaped life in institute and discipline. Though he and Benninghoff both hailed from the Rhineland, their relationship crossed classes and confessions. After Benninghoff’s father left and his mother married a professor and writer, he grew up in the educated, urban, Protestant middle class. By contrast, Spee’s was a noble Catholic family – imperial knights since 1166, counts since 1739 – then prominently represented by his brother Maximilian, the admiral who, having won a famous victory in November 1914, died a hero’s death five weeks later and had a warship named after him. It was a mutiny of sailors in Kiel, the main naval base, that sparked revolution in November 1918, ushering in the Weimar Republic and stripping the nobility of their privileges. Most professors rejected the new order. They defended bourgeois values, but enjoyed hobnobbing with aristocrats. ‘Count Spee’ symbolized their pride and their loss.^14^

**Figure 1:**
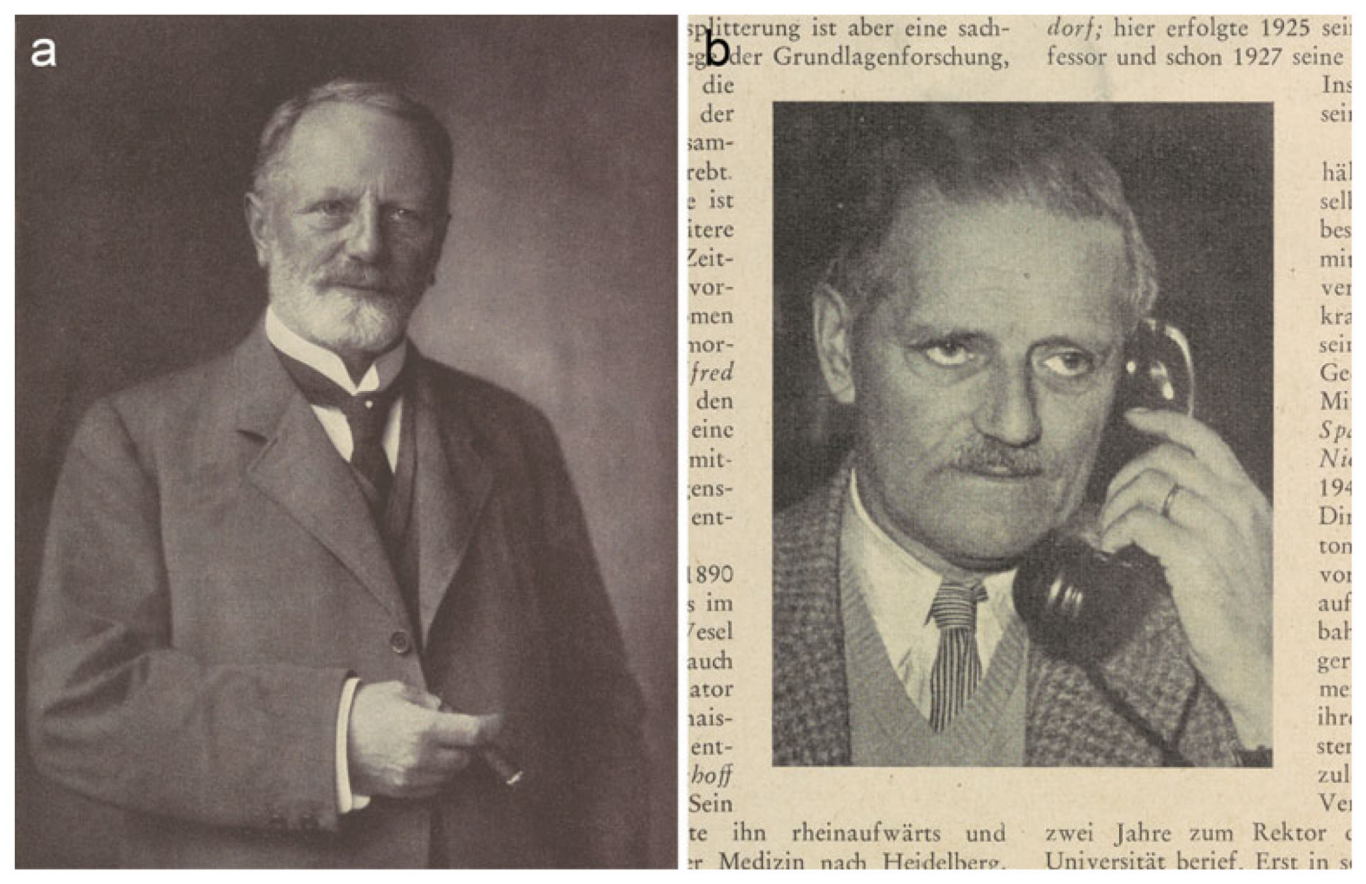
Portraits of Spee and Benninghoff. (a) Photograph of Spee in the seventieth-birthday Festschrift, of which the memoir excerpted the head and shoulders, thus omitting the hand holding a cigar. Frontispiece to *Zeitschrift für Anatomie und Entwicklungsgeschichte* (1925) 76. (b) Obituary photograph of Benninghoff. The phone represents him as a senior, modernizing university administrator; the wedding ring points to his ‘most faithful collaborator’, his wife. From W. Jacobj, ‘Alfred Benninghoff zum Gedächtnis’, *Medizinische Klinik* (1953) 48, pp. 998–999. Cambridge University Library CP304.c.1.78 and L300.b.139.48.

Benninghoff portrayed Spee as a ‘grand seigneur’ and ‘old gentleman’ who performed his class through an ascetic, stoical and self-reliant masculinity (p. 331). This widower with no children had recently bought and moved out to a country estate, Gut Projensdorf. As a seventieth-birthday surprise, he was joined there – with the head of the university, dean of the faculty, professors, and their wives – by Franz Keibel, the reactionary president of the Anatomical Society, and received a Festschrift instigated by Möllendorff (Figure 1a). Many shared Keibel’s esteem for Spee’s solid, uncontroversial research, but the profession surely feted a less than prolific publisher, the former director of a small institute, also as a link to the glory days of empire.^15^

**Figure 2:**
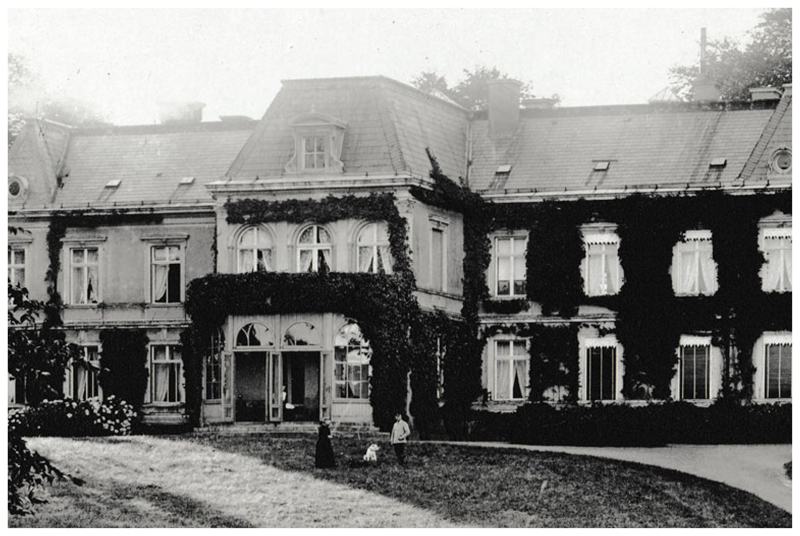
Gut Projensdorf, probably in the 1920s. The photograph is said to show Spee and his housekeeper Mrs Mukatis with a dog in front of the ivy-covered mansion: Britta Gaude, *Altenholz in alten Ansichten*, vol. 2, Zaltbommel: Europäische Bibliothek, 1997, no. 55. Benninghoff asked Spee why he did not sell the estate if it caused him so much trouble; he replied that chasing the pigs from the paddock kept him young (p. 336). Detail from the collection of photographer Marcus Hermann Jansen, courtesy of Gadso Werner, Eckernförde.

Spee could have spent all his time managing his land – not exactly a pastoral idyll, since it lay, however splendidly, beside the Kiel Canal, a busy waterway – but he ‘could not detach himself from his old institute’, and so became, as Benninghoff put it, a ‘wanderer between two worlds fully satisfied by neither’ (Figure 2). ‘He thus developed into a refined figure of an emeritus, who embodied the whole tragedy of this estate in exemplary fashion’ (and all the more so for having no immediate family). Benninghoff depicted Spee as a physical traveller from country to town, who would sometimes appear ‘in an old loden coat and rucksack’, and a mental one between present and past (p. 331).^16^

Benninghoff’s reminiscences drew on ‘13 years’ with Spee and on speeches that had rehearsed his assessment of this ‘unconventional personality’.^17^ Talks, which led one beneficiary to praise ‘a master of the language and also of beautiful thoughts’, prepared the way for prose more literary than any Benninghoff had published before.^18^ He gave an address on Spee’s eightieth birthday in 1935 and spoke at his wake two years later. An oration on leaving for Marburg in December 1940 already covered the main points frankly, including the ‘touching’ way Spee had ‘still lived with’ his ‘things’ and Benninghoff’s own growing insight that no ‘utilitarian approach’ to these objects could do justice to Spee’s long ‘farewell’.^19^

In that emotional leave-taking from colleagues, staff and students, Benninghoff introduced a vivid analogy for the transgenerational ‘community’ that had shared a building. This stepson of the musicologist Willibald Nagel explained that the student saw little of the work that went on ‘behind the scenes’. It

*appears* to him like a sunken *orchestra* from which he only hears the music while he looks at the stage. In this orchestra voices also play which ring out to us from the past. There is in every institute a good *spirit* which somehow inhabits the walls; it sits in the corners or hangs in the air … That is the work produced, the ideas and ideals for which were striven; those are the hopes, and no doubt also the disappointments, which were experienced by our predecessors in this building. And when a researcher has been active in such a building for many years … and one day he leaves it, then … something of the intellectual potential of that work stays behind; what he wanted mixes like an energy with the spirit of the building … and someone who enters … to do new work here … receives … something of this spirit.

Benninghoff romanticized the continuing presence of previous directors, and implied the hope that his own contribution would live on.^20^

If the departed still played in the institute orchestra, then living emeriti more powerfully shaped the experience of the rooms they had built and furnished, and the discussions within. Spee did this most tangibly through the collections, which let him bring ideas and ideals, hopes and disappointments, to life in ways that inspired and amused his younger colleagues. The collections are the focus of Benninghoff’s memorial, which he structured in space as well as in time. Spatially, the memoir follows Spee around the institute, from his room, where the residues of research were a resource for commemorating contributions to knowledge, trying to interest the next generation in rare skills, and even self-diagnosis, to the teaching collections, where he would while away the lunch hours. Temporally, it is punctuated by Spee’s repeatedly engaging his successor in conversation, then dragging him to the microscope to show off his slides. Benninghoff staged these encounters in the order of the publication list with which, like a regular obituary, the memoir concludes.

## Retirement room, microscope and slides

**Figure 3:**
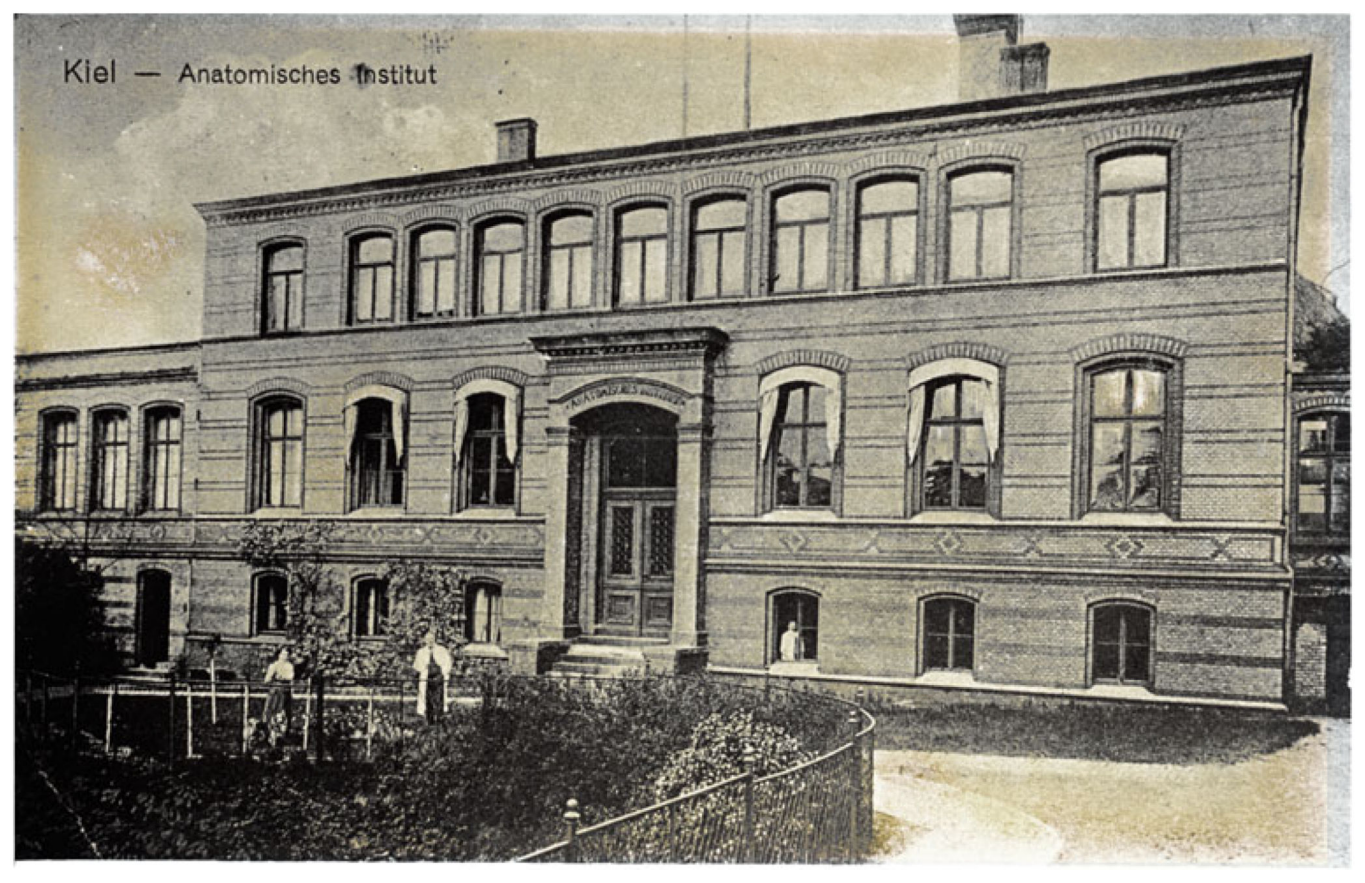
Photograph of a postcard of the anatomical institute in the Hegewischstraße, Kiel, as built by Gropius & Schmieden in 1878–1880, expanded in 1897 and extended further in 1901–1902. The presence of the new top floor and left wing, but not the right wing, date it to 1902–1914. The plain building, with low-pitched arches, was faced in yellow bricks with lines inlaid in red and grey: ‘Zusammenstellung der bemerkenswertheren Preußischen Staatsbauten, die im Jahre 1878 in der Ausführung begriffen gewesen sind’, *Zeitschrift für Bauwesen* (1879) 29, cols. 423–446, 436–437. Anatomisches Institut der Christian-Albrechts-Universität zu Kiel.

**Figure 4:**
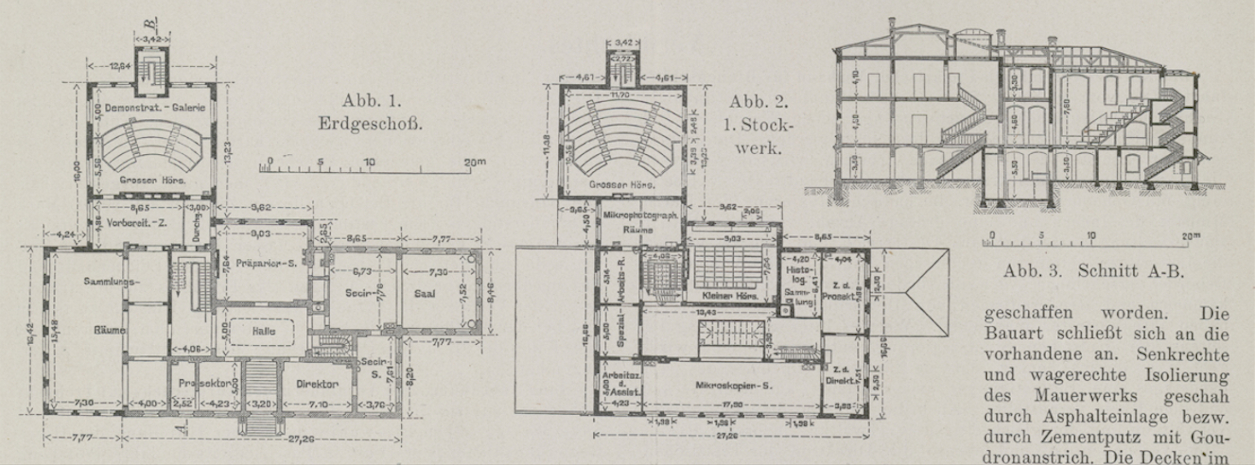
Plans of the Kiel anatomical institute as extended in 1901–1902. Ground (Abb. 1) and first floor (Abb. 2) and section along line A–B (Abb. 3), showing the new left wing with enlarged collection rooms (*Sammlungs-Räume* in Abb. 1) and large lecture theatre (*Grosser Hörs*.) on the other side from the dissecting room (*Secir-Saal*). From Benninghoff’s description, Spee’s was the office marked *Prosektor* in Abb. 1 and his charts were kept in the preparation room (*Vorbereit.–Z*.). From ‘Um- und Erweiterungsbau des anatomischen Instituts der Universität Kiel’, *Zentralblatt der Bauverwaltung* (1903) 23, p. 427. Niedersächsische Staats- und Universitätsbibliothek Göttingen.

The poignant dramatization begins in the secluded room, tucked away at the top left of the ‘wide granite steps’ to the ‘plain but clean building’, on the same lower floor as the collections, which Spee had selected for himself while in office (Figures 3, 4). He kept his research collections in this *Altenteil*, like the cottage reserved for a farmer who has handed over his estate to his son (p. 332).

Benninghoff recalled Spee’s untiring explanations of the advantages of the room, especially the ‘beautiful white-tiled stove’ which was ‘extremely practical’ during the ‘inbetween season’ before the main heating came on. Not that Benninghoff had ever known it lit, and just as well, for it was stuffed with preparations, ‘and it is not certain that anyone would have thought to remove them from their hiding place first’. An old, hard horsehair sofa ‘was supposed to emphasize the dignity of the space’, but ‘was not there for comfort’ (p. 332).

Bleached by the sun, piles of offprints buried a nearby table. ‘One just had to reach out a hand … But it did not happen’, because they were not enticing enough to read. Yet, if ‘put away … they would no longer be so handy. What should one do …? Best do nothing. Fate of many offprints’. Spee wished to keep up with progress, not pan the ‘modern glut’ of literature for gold, and became impatient with authors who lacked his grasp of ‘the essential’. He seemed as solid as the oak furniture that, Benninghoff pointed out, was then standard issue in Prussian institutes. The desk bore ‘the scars of earlier work’ and, next to the microscope, a bowl of instruments. The ‘fine knives and small scissors’ with bone handles ‘dated from a time when practical tools were still decorated and the owner took pleasure in this’ (pp. 333–334).

The old Zeiss microscope ‘dominated the space’, while ‘thousands of preparations … piled up in folders and cupboards’ awaited its power to conjure ‘a new world’ from ‘the unprepossessing patterns on the little glass plates’. Preparing those series of stained tissue slices had obliterated the form as the naked eye perceived it. ‘To see a new world’, Benninghoff suggested, ‘we must destroy the old’ – perhaps a veiled acknowledgement that the price of progress had to be paid. This posed the synthetic challenge of bridging between the microscopic and the macroscopic, or more precisely between as many worlds as there were levels of resolution – a popular theme of reflective microscopists and something Benninghoff had attempted in his own research. He could understand Goethe’s mistrust of spectacles as leading the wearer to overestimate his judgement. Spee, having experienced the ‘triumphal march of microscopical technique’ through the late nineteenth century, was more trusting. He even tested his senses on the instrument. Once he asked Benninghoff if the image was dull or clear, and then again after he had ordered new lenses from Zeiss. This convinced Spee that he had a (then inoperable) cataract; ‘he never consulted an ophthalmologist’ (pp. 334–336).^21^

‘The old count thus had all his microscopical treasures … around him’; that is, the slides that he had accumulated in the course of his research and made for teaching. He did not like to lend the latter, although the institute initially had no others and he had done ‘hundreds of beautiful drawings’ to elucidate them. Trying to understand this frustrating behaviour, Benninghoff reported Spee’s view that only he could explain ‘the finer points’. ‘When he died … many preparations … became worthless; they were nothing without the count.’ As if to forestall that event, Spee engineered conversations that Benninghoff spun into a survey of his research career. For the early work in embryology Benninghoff deferred to a brief article that Möllendorff, who researched cells and embryos, had written for Spee’s seventieth birthday, but embellished the account by recalling personal interactions (p. 336).^22^

Spee ‘often stood’, bolt upright, ‘at the door of his room, waiting but not asking; he wanted to show us his treasures’. There ensued a greeting, a powerful handshake and an exchange about trouble on the estate that drew Benninghoff in, until the two men were near the microscope. Spee would then ask, ‘By the way, have you seen my preparations of the implantation of the guinea pig egg?’ He fetched a large carton, to which a series of stuck-on photographs provided a guide, took slides out of their folders and put them on the stage. The osmium staining mimicked pen-and-ink drawings, so ‘excellent’ was the technique that had ‘rubbed off’ on Spee from Flemming, describer of mitosis (pp. 336–337).

Benninghoff’s Spee innovated conservatively in descriptive embryology by applying methods he learned from Flemming and that other local master, Hensen. Spee’s oldest trophies dated back to the doctoral work on guinea pig development that Hensen, a physiologist unusual in his embryological interests, had supervised in the early 1880s. The topic had been controversial for three decades, since the pioneer of mammalian embryology, Theodor Bischoff, claimed that guinea pigs deviated from the norm he had established for rabbits, dogs and deer. Supporting Hensen’s critique, Spee showed how artefacts of post-mortem degeneration had misled Bischoff, and worked out the development so well that it helped researchers grasp human implantation. Though the count did not want his hard-won experience to go to waste, his juniors never did take up his offers to show them his refinement of Hensen’s tricks for collecting guinea pig eggs (p. 337).^23^

**Figure 5:**
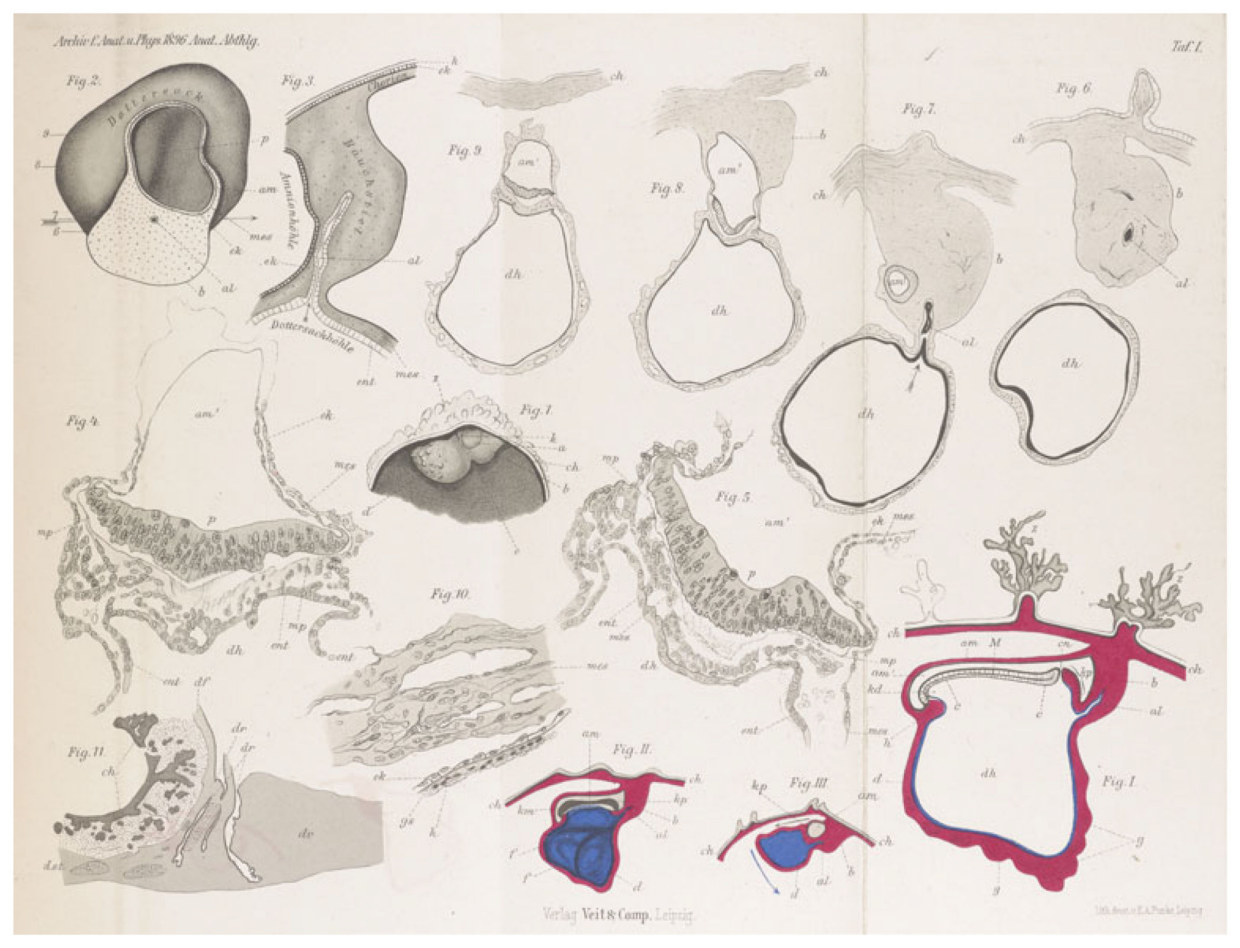
Spee’s drawings of the early human embryos ‘Gle’ and ‘v.H.’. The plate shows semi-schematic median section and profile views of Gle (figs. I–II), and various views and sections of v.H. (figs. III, 1–11); figure 2 is based on a model. From F. Graf v. Spee, ‘Neue Beobachtungen über sehr frühe Entwickelungsstufen des menschlichen Eies’, *Archiv für Anatomie und Entwickelungsgeschichte* (1896), pp. 1–30, Plate I. Cambridge University Library Q304.c.11.39.

Spee’s colleagues did let him tell the suspenseful story of those analyses of human embryos which in the late 1880s and the 1890s had grounded his more independent ‘fame as a connoisseur’ (Figure 5). Among the embryologists competing to describe the youngest preparations, any of two or three weeks counted as valuable rarities. Spee spoke of the tension as he opened the egg chamber, when ‘one misplaced cut’ could have ‘ruined everything’. He reminded his listeners that he had insisted on direct sunlight, and of the trial of patience as he waited for cloudy northern skies to clear. Then he displayed the models he had cast in plaster and painted (after the wax originals he reconstructed from the serial sections); every textbook and handbook of embryology carried at least one illustration (p. 337).^24^

Another preparation, an embryo preserved in spirits, Spee ‘took out with special care and showed like a precious stone. It was somewhat damaged. “[Aleksey Nikolaevich] Severtsov did that, the swine”, he would then always say. Full of life, his eyes still flashed when he showed a beautiful preparation or saw a beautiful woman’, Benninghoff testified, with the equivalence perhaps presenting the former as a conquest of nature (p. 339). Possessive love prevented productive appropriation, as of the slides that might have been used in teaching. Benninghoff’s Spee also appears unconcerned about preserving his human embryos for posterity; he would have been more likely to deposit them in a central collection had he worked in the United States.^25^ But these demonstrations prompted conversations that pressed ancient claims and kept projects alive. For Benninghoff, they illustrated how much had been achieved with simple methods, decorated tools and the right approach.

## From physiological anatomy to functional systems

Scientists’ primary loyalties have been to their disciplines and approaches within them. Benninghoff engaged with Spee’s research collections in the anatomists’ house journal to portray him as a forerunner of his own agenda and to teach its correctness. In studies of cartilage Benninghoff had moved from comparative anatomy to a more functional orientation to organic form before arriving in Kiel, and in dialogue with anatomists who had worked elsewhere: Wilhelm Roux, the founder of ‘developmental mechanics’, and Hermann Braus, whose revolutionary textbook paved Benninghoff’s way. But as director Benninghoff made ‘functional systems’ the programme for the Kiel institute’s work and promoted it through the textbook first published during the Second World War and still in print.^26^ With a secondary allegiance to his universities, it suited him, as he traced the expansion of Spee’s interests into adult anatomy, to place his own research within a local tradition.

‘It is winter and there is a cold wind outside. The count comes into the institute completely frozen through … “Damned cold today … and Projensdorf was badly heated … It is nice and warm here. By the way, have you seen my preparations of the intestinal villi?”’ In 1885 these had earned Spee his habilitation, the second doctorate that in general gave the right to teach and specifically let him transfer to the anatomical institute two years later. But he stayed so ‘faithful’ to Hensen’s physiological programme that Benninghoff imagined the field of his qualification as the unusual ‘embryology and physiological anatomy’.^27^ Spee had introduced a dynamic perspective with the concept of a ‘villus pump’, the rhythmic contraction that aided the flow of blood and of chyle. According to Benninghoff, anatomists embraced the idea straightaway; physiologists rediscovered the pump forty years later; and it was widely accepted once it could be seen on film. He thus advocated a functional approach within anatomy, pressed his discipline’s priority claim and chided his generation for their dependence on moving images (pp. 337–338).

The count, Benninghoff related, had not been to the institute for a while. He had had an accident and lost some front teeth, a loss he found hard to accept. Spee raised with Benninghoff the possibility of transplanting tooth buds, and they discussed which species might make the best donor; a piglet perhaps, ‘but not everyone would want to have pigs’ teeth grow’.^28^ This triggered Spee to affirm his faith in a junior colleague’s work on tooth development. He would show Benninghoff the preparations. ‘And again we were standing in front of the microscope.’ Though Benninghoff was in a hurry, Spee took several attempts to find the best place. From here the conversation moved on to other dental matters, including the investigation that led to the description of ‘Spee’s curve’, the arrangement of the chewing surfaces of the molars in a line rising towards the back of the mouth (pp. 338–339).

**Figure 6:**
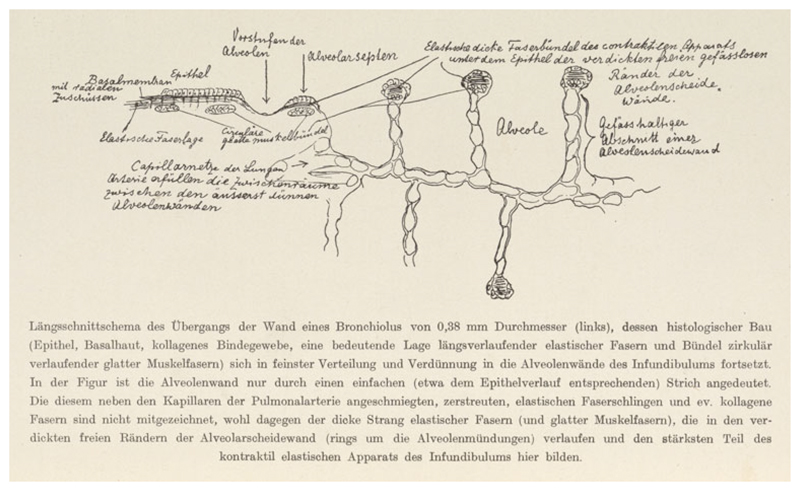
Spee’s hand-labelled diagram, in his article on lung preparations, of a bronchiolus (in longitudinal section) dispersing and thinning into alveoli. He emphasized the contractile apparatus, the bundles of elastic fibres and smooth muscle, as well as the thin walls. From Graf F. Spee, ‘Zur Vorweisung von Präparaten menschlicher Lungen, die in natürlicher Spannung konserviert wurden’, *Verhandlungen der Anatomischen Gesellschaft* (1928) 37, pp. 302–306, 304. Royal Society of Medicine Library.

Benninghoff presented Spee as pioneering investigations of functional questions that others were just beginning to tackle, and as remaining absorbed by them to the end. His last, short publication had been about the effects of tension on the microscopic as well as the macroscopic appearance of the lungs. Benninghoff described this research as having started in good anatomical fashion with ‘unusually beautiful preparations and sections of the thoracic cavity’ (p. 340). He did not spell out, but Spee had told his colleagues, that the beauty relied on a departure from the usual practice of studying lungs that had contracted to a third of their volume after removal from the thorax. Instead, Spee had frozen the cadavers of people executed by beheading, sawn them up and kept the elastic fibres stiff by allowing the slices to thaw in a solution of mercuric chloride. This avoided the chaos resulting from what was misleadingly called ‘collapse’, and revealed the ‘wonderful order of all the parts’, even the thinnest-walled (Figure 6).^29^

**Figure 7:**
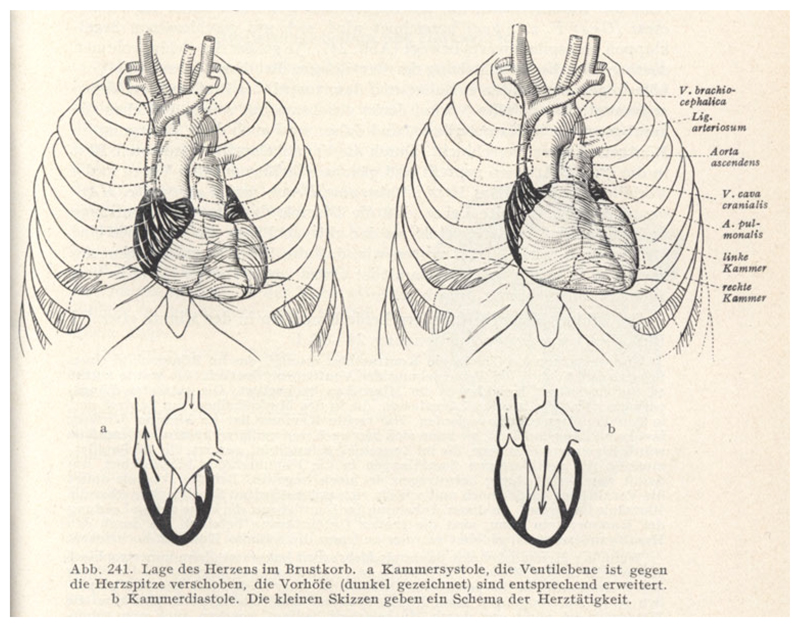
Diagrams of the ‘Position of the heart in the thorax’, at (a) ventricular systole and (b) diastole, through which Benninghoff’s textbook introduced students to Spee’s concept of the valve plane. In systole, ‘the valve plane is displaced towards the apex of the heart, [and] the atria (drawn dark) are expanded accordingly’; they thus suck in blood from the veins, as shown in the schematics. From Alfred Benninghoff, *Lehrbuch der Anatomie des Menschen: Dargestellt unter Bevorzugung funktioneller Zusammenhänge*, vol. 2, part 1: *Eingeweide*, Munich: Lehmann, 1942, p. 421.

Benninghoff explained that similar preparations had inspired Spee’s functional reflections on the movements of the heart. Reviving the notion that this organ is a suction pump as well as a pressure pump, Spee had described the motion of what he christened the ‘valve plane’. The ventricles become smaller at systole not only widthways, but also longitudinally, because the valve plane – the floor of the ventricles with all the ostia (openings) – moves towards the apex, stretching the atria and sucking in the blood (Figure 7).^30^ Benninghoff had himself observed the process in living salamander larvae and had a student shoot a film which, despite his pose of regarding the medium as superfluous, he elsewhere judged had ‘brilliantly confirmed’ Spee’s and others’ observations.^31^ Here Benninghoff reported the count’s lack of surprise on seeing the movement for the first time; that inspired anatomist had deduced it from dead preparations. But Spee was full of fresh thoughts, such as about why the apex stayed still when the valve plane moved. ‘He often pulled me into his room with the words: “I must tell you … more about the displacement of the valve plane.” Then the eyes lit up in his old face, already somewhat lost in reverie, and like an old magician he began to talk and to draw’ (pp. 340–341).

Benninghoff summarized Spee’s research as having sought to advance ‘from a static to a dynamic perspective’. By respecting the ‘natural interrelations’ of the parts, Spee’s descriptions led him to consider function: from villus anatomy to the pump, from lung topography to pressures, and from positional relations in the heart to the movement of the valve plane. Benninghoff surprised Spee by stating this in a speech for his eightieth birthday; the count had taken it for granted. But when Benninghoff wondered if it were mere chance that the next Kiel generation had extended the approach as ‘functional systems’, Spee responded, ‘Who knows … perhaps it is the genius loci’, or spirit of the place (pp. 341–342). Spee seems to have encouraged Benninghoff’s conviction that there was such a thing.

## Conducting a few bars

Benninghoff followed Spee as he left the room and his research materials to visit the teaching collections. His successors had taken these over when he retired, but since Spee was invested in the contents – which, as director, he had enriched at the expense of his own research – the stage was set for clashes between his interest in memory and theirs in modernization. Though personal research collections might document past discoveries, professors were supposed to keep their teaching up to date.

Benninghoff continued to place the count in a building occupied by people and things, and sensed through ears and hands as well as eyes. Spee had once won the ‘love and admiration of his pupils’ through his own obvious ‘love of the subject’ and ‘happy dedication to the task’.^32^ Now he went to the classrooms while the students were at lunch. One who stayed behind reported that ‘an old gentleman with a white beard’ had come into the dissecting room. ‘But he knew a tremendous amount about the knee joint’ (p. 342).

After the ‘noisy building’ had ‘loudly discharged its visitors’ it became ‘very quiet’. ‘Then came his time, when everything belonged to him again; then he softly began his lonely walks.’ Engrossed in his subject, the ageing genius was lost to the world and his colleagues had learned not to disturb his thoughts (p. 342). Entering the lecture hall (Figure 4), Spee wandered among the charts, blackboard drawings, preparations and models, an experience for which Benninghoff invoked his musical analogy. The farewell speech had implicitly presented himself as the conductor, proud to have created a ‘community’ in ‘harmony’.^33^ The memoir romanticized the anatomist as artistic director:

It is like a glance into the empty orchestra pit after a concert. The stands are still in position with the sheet music open. If a musician pokes around among the notes he can still experience something of the concert that recently faded away. In this way in the empty lecture theatre the count may in his thoughts have conducted a few bars of the lecture he had formerly given himself. (p. 343)

Like a conductor with a score, a lecturer’s performance brought objects to life. The anatomy teacher revivified relics of the dead – but only for his allotted time. After a concert, after a morning’s teaching, after a career: sound and bustle gave way to quiet emptiness and silent reflection.^34^

Spee also haunted a room behind the lecture theatre where his teaching charts hung (Figure 4). ‘What anatomist’, Benninghoff asked, ‘has ever drawn all lecture charts himself?’ Spee had produced many ‘original’ views with an equally ‘original’ technique he often recommended in vain: softening the lines from coloured crayons with linseed oil. (Though the medium is different, Figure 6 might give some sense of how they looked.) The collection was incomplete when retirement interrupted the work. His successors put the plates aside as too small for the expanded classes, but they ‘stood beyond any question of mere utility’. No one dreamed of disposal and Spee never asked why they were not used. He would take one out and contemplate it at length. ‘[T]he spirit of his past life … inhabited [these plates] as a mute force that could no longer be released. Yet the count stayed among his favourites … let his memories come to life again and took his … long farewell’ (p. 343). Where the speech had stressed the mixing of a researcher’s ‘energy’ with the institute’s ‘spirit’, the memoir also thematized the failures of communication that presaged meaning loss.

## Among his old things

In Benninghoff’s telling, these solitary rounds moved on to the macroscopic preparations in the anatomical collection, which had been more contentious between Spee and Möllendorff than the slides or the charts (Figure 4). Dealing with the traumatic episode took all Benninghoff’s diplomacy, but was eased by the passage of time and the principals’ deaths. He developed his tale of the foibles of the emeritus while narrowly avoiding ridiculing a man for whom he and his junior colleagues felt genuine respect.^35^

‘Time and again’, Benninghoff recalled, Spee ‘would bend his white head over the preparations as if he were looking for something. He knew every single piece and had made many himself’. There were ‘numerous excellent and elaborate representations’ and others which – in accord with ‘his artistic nature’ – ‘were only sketched’. Spee looked after this collection ‘as if he could continue working on it forever and such a project ever be completed. He really could not hand it over.’ His research materials represented studies that publication had provisionally concluded, but the institute collections, with their aspiration to be comprehensive and current, were a constant work in progress. It hurt when ‘a new director came’ and (Benninghoff used the passive voice) ‘the collection was reorganized and some things that appeared unusable removed’. Distancing also by generalization, he explained that old directors no longer saw problems, but the new saw too many; among the latter, ‘passionate organizers’ let ‘the means become the end’ (p. 344).

**Figure 8:**
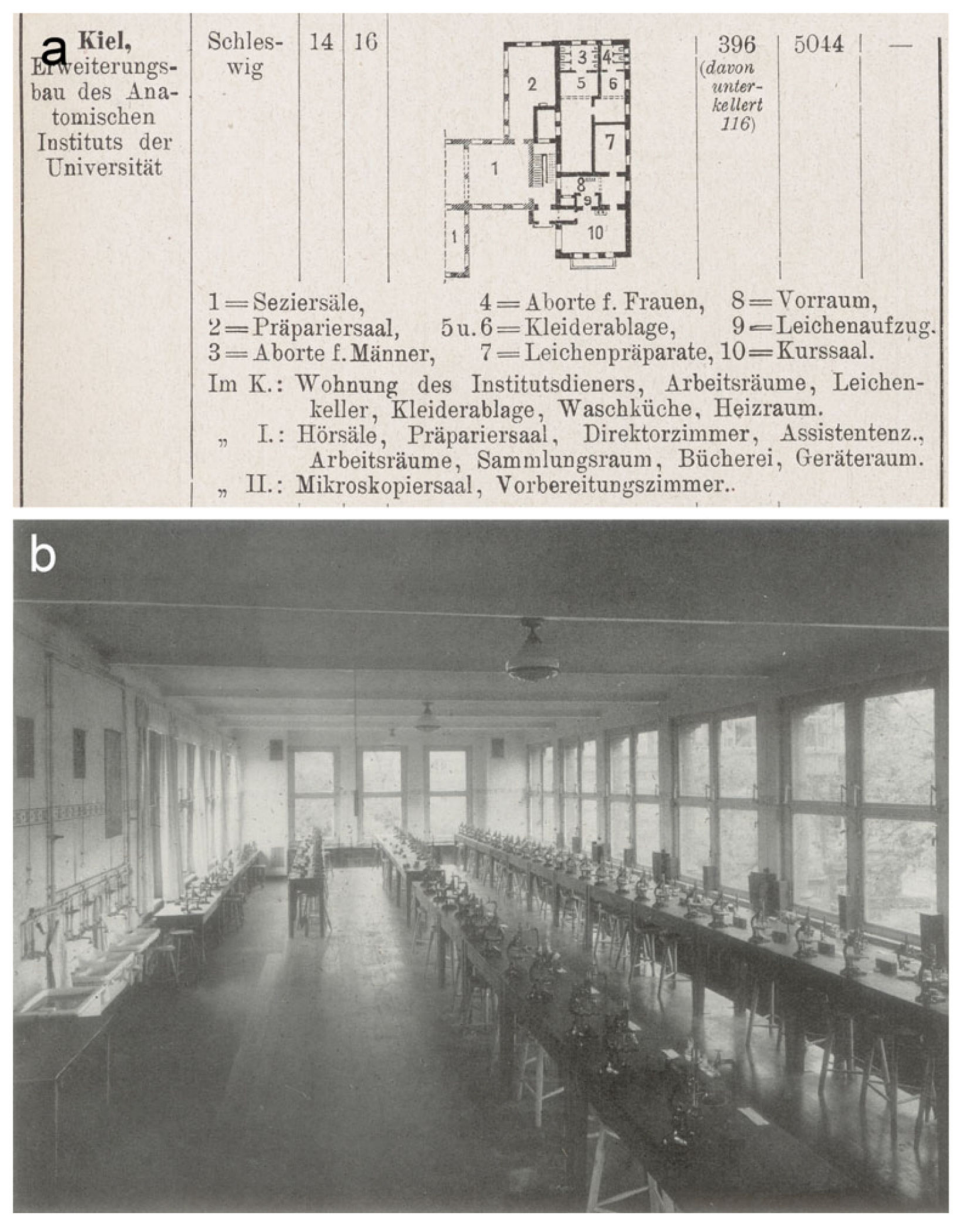
The two-storey extension that in 1914–1916 doubled the size of the anatomical institute and allowed three hundred students to be taught simultaneously. (a) Plan of the ground floor, with dissection and microscopy rooms listed for first and second floors respectively. Part of table from ‘Statistische Nachweisungen betreffend die in den Jahren 1915 und 1916 unter Mitwirkung der Staatsbaubeamten vollendeten Hochbauten’, supplement to *Zeitschrift für Bauwesen* (1918) 68, p. 7. Cambridge University Library T402.a.21.54. (b) The microscopical classroom on the top floor, after refurbishment in 1926. The physiological institute is just visible through the large window facing north-north-east. From Kurt Feyerabend, *Die Universität Kiel: Ihre Anstalten, Institute und Kliniken*, Düsseldorf: Lindner, [1929], p. 36; see also the site plan at p. 15.

Spee was proud of having expanded the building to accommodate more students. As prosector he had maintained and enlarged the collections and helped Flemming during a major extension in 1901–1902 (Figure 4). As director he oversaw a two-storey wartime addition that doubled the institute size and in 1921 was described as ‘uniquely beautiful and functional’. Students benefited from ‘[e]xcellent hygienic and lighting arrangements’ as well as ‘extensive demonstrations’ from the ‘rich’ anatomical museum (Figure 8).^36^ In 1925 Möllendorff praised Spee as an ‘extraordinarily beloved teacher’ who ‘sacrificed all his time’ for pupils he inspired through ‘the magic of his personality’. He had made the institute a ‘magnificent site of teaching’.^37^

It is almost shocking to discover that the previous year Möllendorff had had the ministry told that ‘under its previous director’ the anatomical institute ‘was without a doubt the weakest point in the medical faculty’.^38^ New directors often asked for money by branding as obsolete what long-serving predecessors once hailed as state-of-the-art,^39^ and several challenges were evident in Kiel. Belt-tightening during the war and the inflation had combined with a further increase in students to render the facilities inadequate; the latest extension was still not properly furnished. Spee had supported the establishment of an anthropological department at the expense of the parent discipline. No histological technician or preparator was currently employed. But Möllendorff’s complaints went further than this.

The new director targeted the absence of a workshop or demonstration stands, furniture or equipment; the presence of microscopes so primitive that Leitz no longer made them for schools; and a library that had acquired almost no books, stopped taking several important journals and done little binding since Spee took office – but the litany began with the collections. This ‘foundation on which … teaching and research … must build’ was in Kiel ‘in many cases in ruins’ and ‘unsystematically arranged’. Teaching could hardly proceed, preparations and models were so scarce. The latter mostly dated from the nineteenth century; Spee had bought just two since 1908. The most important macroscopic models were missing or wrong, and the majority of the Zieglers’ ‘famous and absolutely indispensable’ embryological waxes were also lacking. A few wet preparations were ‘superb’, but ‘the overwhelming majority … is in the process of disintegration’. This was a consequence of the mode of storage (all could be removed from the glass for demonstration), insufficient alcohol and simple ageing. ‘[F]or many topics’ ‘no good preparations could be shown’.^40^

Like other anatomists, Möllendorff expected that teaching preparations would be replaced as they wore out. His reference to storage practices relates to a little-remarked trend in their institutes to supplement collections that served for display and demonstration, and dedicated ‘handling collections’ which were passed around during lectures, with ‘learning collections’: selected preparations and associated visual aids that were housed in rooms set aside for students to study the objects at leisure and thus fix the structures in their minds. For this purpose, Möllendorff had begun remounting items in glass jars with parallel (not tapering) sides and closed with putty.^41^ The institute was overhauled in 1926 and the anthropologists left.^42^

The changes ‘disturbed’ Spee. According to Benninghoff, ‘He could no longer find his way around’ and missed such favourites as an embryonic brain. ‘We thought nothing of it and had removed it, but for years the count asked where it was and much regretted its disappearance. He had based a special theory on it.’ In a draft, that sentence had less tactfully continued ‘and not noticed that through the passage of time or under the pressure of the theory the preparation had almost disintegrated’.^43^

Spee still lived among his old things and, when his former technician accompanied him, ‘the old time was restored’; ‘he governed his collections in his mind again and surrounded them with that love which feels itself kin to the formations of nature’ (‘formations of madness’ in the draft).^44^ Spee would take preparations out, reawaken memories and say goodbye. Though aware of ‘the fragmentariness’ of such an enterprise, he sought in vain to bring some kind of conclusion to his life’s work. ‘Every farewell is painful, but the late one is tragic’ (pp. 344–345). He could neither finish the collection nor let go.

Spee remained alert, but age shifted his interest ever more pleasantly to the past. Benninghoff concluded his reminiscences with final iterations of the request that punctuates them throughout, and now directly addressed the impending end:

‘Have you already seen my lung preparations?’ [Spee] asked and drew himself up. ‘No, Count, I don’t know them yet.’ And he showed them to us. After some time he came again with the same question and now we told the truth: ‘Yes, Count, we have seen them already.’ When in the last years he asked again and again: ‘Have you seen my lung preparations already?’ then we said: ‘No, Count, we have not seen them yet’, and he showed them to us – emeritus. (p. 345)

The double reversal – Benninghoff’s acknowledgement that he had avoided Spee’s demonstrations, then learned to accept them – heightened his show of empathy and helped to generalize Spee’s predicament.

## Anatomical politics

Anyone who knows a retired academic will recognize the repeated storytelling, but Benninghoff’s memoir, published in the *Anatomischer Anzeiger* (Anatomical Gazette) in September 1944, is also a specific product of German anatomy towards the end of the Second World War. It should be considered in relation to the politics of memory and the ethics of collecting under and after National Socialism.

Historians have found the ‘unpolitical’ Benninghoff difficult to place. They tend to agree that this moderate conservative unenthusiastically went along with Nazism, while keeping his distance, though some point out that he may have gone further in a few respects.^45^ In the Kiel medical faculty between 1935 and 1940 he served as deputy to an inexperienced Nazi dean, smoothing the way yet having his career blocked by a denunciation as ‘liberal, friendly towards Jews, and Catholic’. (He was Protestant.)^46^ Speeches and lectures to colleagues and students endorsed those aspects of the ‘new world view’ that chimed with his core commitments to nationalism and holism. In a 1934 lecture at a training camp and in addresses to medical students, Benninghoff celebrated ‘the great discovery that the *Volk* is also *an organism*’ which could become sick. Treatment should distinguish between ‘biologically valuable and inferior parts’, and he praised the 1933 Sterilization Law as exemplary. Yet few German medics would have disagreed and, unlike more right-wing holists, Benninghoff did not, to my knowledge, discuss race.^47^ He argued that ‘science does not float free in space’, but ‘is a child of its *Volk* and its time’, while crediting the rise of holism to independent developments in various fields rather than a ‘political diktat’.^48^ He publicly demonstrated respect for Göppert, who was Jewish, at the Anatomical Society congress in 1938 and through their collaboration on his book.^49^

After the war Benninghoff complained, with some evidence, that he had been forced to stay in a small university until 1941, when it suited the state to replace him with his junior colleague Enno Freerksen, an active Nazi and SS man. Benninghoff enrolled in the NSDAP on his appointment to direct the Marburg institute, but only, he claimed, because Freerksen handed him an application to sign in the presence of staff members and he wished to safeguard his ‘life’s work’.^50^ Yet when pleurisy consequent on tuberculosis necessitated sanatorium treatment in August 1942, Benninghoff went to a Swiss institution, the Deutsches Haus (German House) at Agra, south of Lugano, where Nazis ruled the roost.^51^ He had come to an accommodation with the regime, but it still seems unlikely that he who once joined in German jubilation over the annexation of Austria had developed any deeper commitment as the dictatorship celebrated its last military victories.^52^ That same year the lecturers league told the Party chancellery: ‘He will probably never be a National Socialist out of innermost conviction. He holds as a matter of principle the for us impossible view that fundamentally science and politics have nothing at all to do with each other’.^53^

The year-long convalescence in Switzerland could have given Benninghoff, who had been busy with his textbook and with moving to Marburg, the leisure to write his reminiscences of Spee.^54^ In autumn 1943, he showed a draft to a friend; Friedrich Griese, a former Kiel headteacher and one of the most successful ‘blood-and-soil’ novelists under National Socialism, was struck by ‘how beautifully you … tell a story’ (and pronounced himself unable to improve anything of substance).^55^ By this time, defeat at Stalingrad had soured the public mood and Switzerland provided access to ‘the foreign press’.^56^ This presumably reinforced Benninghoff’s scepticism and helped prepare his post-war stance.

By 1947 Benninghoff reckoned that he had engaged in ‘ongoing active resistance’, given the limitation that ‘I … represented a completely unpolitical field and my profession did not directly give me the opportunity to carry out oppositional actions.’^57^ He was classified merely as ‘exonerated’, but in fact joins the many scientists who gave the regime qualified support, let their work justify silence in the face of injustice, and then signed letters to denazify others who had done worse. A senior figure after the war, he was on the German Research Council and headed the University of Marburg for two years (Figure 1b). In a speech to new students he now explained that ‘Today’s student … acts continually in a political space, perhaps without realizing it and often unaware.’ Some rejected politics, but ‘even this rejection is fundamentally a political act’^58^ – and so it had been in anatomy.

The Nazis had changed the discipline by dismissing Jews and political opponents, and by providing ever more cadavers for dissection from people executed for resistance, defeatism or the trivial thefts that became capital offences. Anatomists made the most of their unusual access to the freshly killed bodies of young people, and a few designed experiments around the judicial murder of the ‘future dead’.^59^ Benninghoff’s memoir is silent on the ethics of collecting; he likely saw no ethical issue, and none was directly implicated in the relations between the two men, for all Spee’s interest in cadavers of the beheaded.^60^

**Figure 9:**
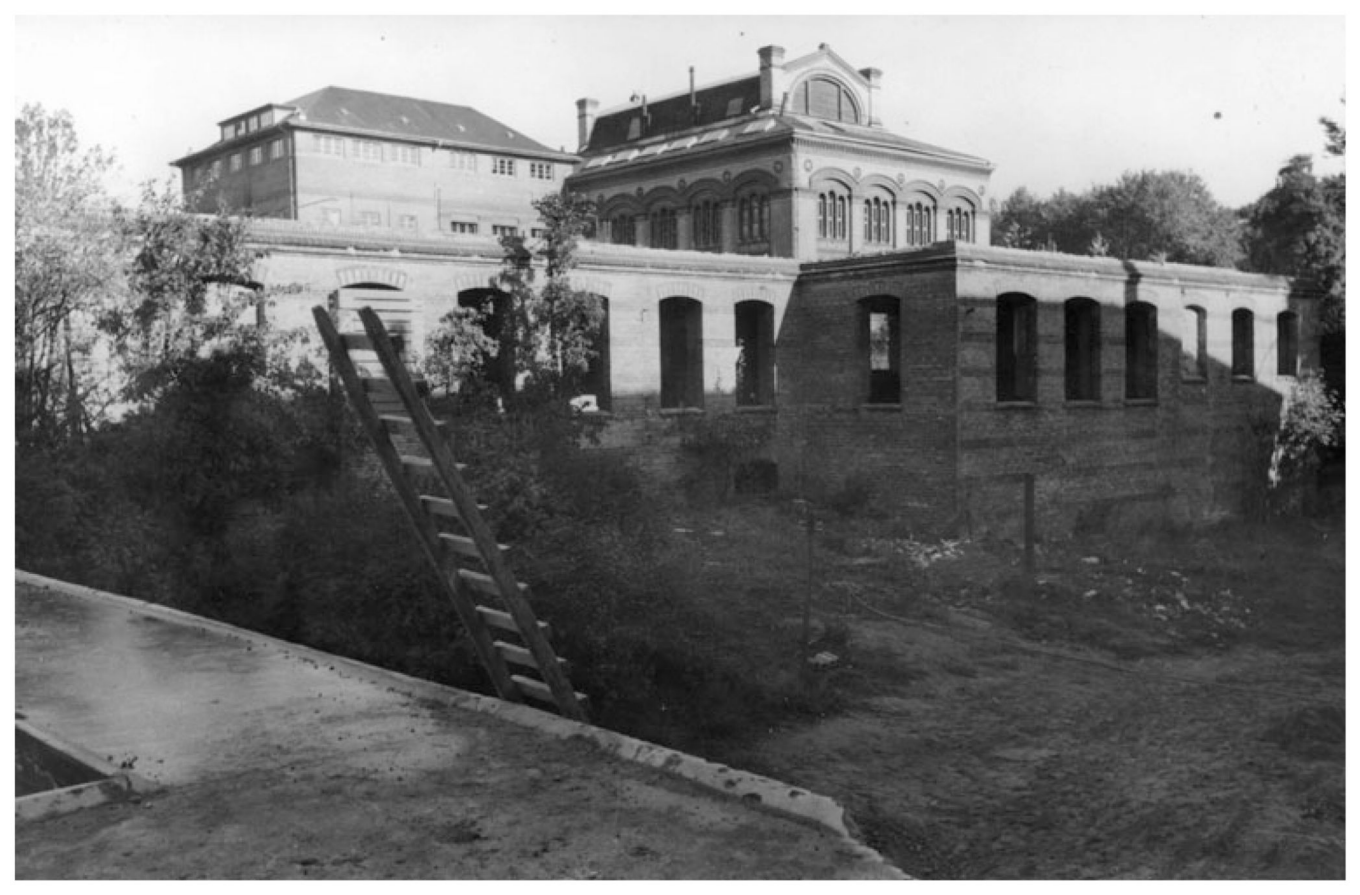
The ruin of the old anatomical institute (foreground), photographed from the back around 1950. Stadtarchiv Kiel, 1.1 Fotosammlung 72735.

Benninghoff did mention that Spee’s charts had been destroyed by fire when Allied bombs hit the left wing of the building with the large lecture theatre in April 1942 (p. 343). Firebombs demolished the rest of the institute a month before the article came out (Figure 9).^61^ Anatomy in the ‘City of the Imperial Navy’ was thus cast as a victim. Yet among the seventy-four cadavers left in the formalin baths in the ruined basement when Germany capitulated were those of six resistance fighters, delivered to the institute under Freerksen. Interred without coffins in a mass grave in June 1945, only pressure from relatives forced their reburial with honour. Freerksen’s successor, Wolfgang Bargmann, blocked inquiries while seeking to have the bodies exhumed for teaching. Until recently, no one accorded the other sixty-eight, branded ‘criminals’, much respect.^62^

Benninghoff did comment implicitly on his colleagues’ treatment of one another. The liberal Göppert had complained that Wilhelm Lubosch, also Jewish, was excluded from his institute and forbidden to take his preparations.^63^ Benninghoff would advise one of the more compromised anatomists, fighting for access to his research collection, that – based on the precedents of Flemming, Spee and Möllendorff – it was his own.^64^ Himself over-tolerant of Nazism, Benninghoff offered a model of tolerance in relations between professors that gained force by the stark contrast with the ways others had behaved.

Benninghoff joked that the Spee obituary was his most successful publication, a claim supported by a few letters thanking him for offprints.^65^ The Reich chief of medical science, the surgeon Paul Rostock, claimed to have read the memoir ‘word for word’, but showed more interest in Benninghoff’s extending some animal research on scar tissue to human beings.^66^ The primary audience shared memories of the Kiel medical faculty. Gynaecologist Robert Schröder, a Party member since 1933 who moved to the large University of Leipzig in 1936, reported his ‘greatest joy and pleasure’ at the ‘charmingly kind way’ Benninghoff brought out ‘the idiosyncrasies of the strange old count. I experienced him similarly and every time he got hold of me he brought the conversation in the twinkling of an eye onto a scientific topic that interested him just then. – For me the Kiel years were the best’.^67^ This was a common refrain about the ‘climate of work and of friendship’ in a small but growing and busy university with close personal ties.^68^ The physiological chemist Hans Netter asked Benninghoff twice if he could spare a copy of the ‘little book’ for ‘an ancient Kiel man’. (Netter had qualified there in 1923 and stayed on.) ‘[S]o he can derive pleasure from it, because they say one can do that, and I … need … such pleasures in this time, which is not entirely stress-free’.^69^ Described as sensitive and unpolitical, Netter was pressured into the SA in 1933 and joined the Party in 1937. Research for the air force led him into situations, notably participation in a conference at Dachau concentration camp about lethal seawater experiments (of which he is said to have disapproved), that led to his temporary removal from office after the war. His institute had been flattened.^70^

At a time of careers variously truncated, interrupted and wrecked, opportunistic and murderous, and of collections damaged, ruined and stolen, Benninghoff wrote a requiem for an era when a retired professor could linger with his things. His demonstration of sympathy towards Spee set a positive example, and he rebuked the careerists by presenting the count as driven by love. The piece is apologetic by omission, nevertheless. His colleagues found in it an escape from harsh realities that may have helped them come to terms with loss without facing their own complicity.

## Conclusion

Benninghoff, who died, much mourned, in 1953, aged sixty-two, was never an emeritus, but left a vivid memorial of how a retired professor influenced intergenerational relations through his collections. Written in the shadow of Nazi brutality, these reminiscences came out of the German academic culture of deference and of age limits introduced in the Weimar Republic. Yet they direct attention more generally to the importance of retirements as periods, not moments, of vulnerability in the histories of collections. Take the controversial Göttingen anatomist Erich Blechschmidt’s famous human embryos. When he retired in 1973, he treated as private what his successor and his former employer had regarded as state property, and took home thousands of microscope slides; he sent many to the United States. Only after long negotiations, including with Blechschmidt and his widow, were the slides reunited in the institute.^71^ The cases must be legion; where Benninghoff’s memoir is special is in its dramatization of the emotions involved.^72^

Retirement drains power, but Benninghoff’s Spee still shaped the meanings of his belongings and through them institute life as well as assessments of his own career. Spee retained complete control over the research materials with which he pushed colleagues to engage. He demonstrated slides at the microscope, occasioning conversations, also about drawings and models, that kept him in touch and his discoveries in their minds. He failed to pass on his embryological skills, but succeeded in having Benninghoff present his physiological anatomy as an inspiration. Spee refused to lend his demonstration slides. Yet having lost authority over the institutional collections, he had to watch his successors remove, reorder and remount objects that remained full of significance for him, though they felt obliged to keep his charts. Benninghoff made amends by preserving for posterity some echo of Spee’s life among his old things.

The memoir explores the relations between scientific generations, those players in a musical community, as mediated by collections in decline. Collectors and collections often age comfortably together, and the default state – gradual decay, even disintegration – did not trouble Spee, though the self-diagnosis of his cataract brought unwelcome news of fading faculties. His collections represented achievements and insights that underlay and went beyond his published work, and he tried to stave off the inevitable loss of meaning. Sometimes his things evoked memories of teachers and provoked responses from pupils and successors. At other times, possessive love hindered use or Spee brought items to life in performances that, like a conductor without orchestra or audience, only he could hear.

## Footnotes

1 A. Benninghoff, ‘Erinnerungen an den Anatomen Graf F. von Spee’, *Anatomischer Anzeiger* (28 September 1944) 95(15/20), pp. 331–347. References to pages in this work are given parenthetically in the text.

2 On obituaries see, for example, Dorinda Outram, ‘The language of natural power: the “éloges” of Georges Cuvier and the public language of nineteenth century science’, *History of Science* (1978) 16, pp. 153–178; on memory in German anatomy, Nick Hopwood, ‘A marble embryo: meanings of a portrait from 1900’, *History Workshop Journal* (Spring 2012) 73, pp. 5–36; and Sabine Hildebrandt, *The Anatomy of Murder: Ethical Transgressions and Anatomical Science during the Third Reich*, New York: Berghahn, 2016, pp. 258–295; on physical spaces, Simon David John Chaplin, ‘John Hunter and the “museum oeconomy”, 1750–1800’, PhD dissertation, King’s College London, 2009, pp. 174–214; and on emotions, Samuel J.M.M. Alberti, *Morbid Curiosities: Medical Museums in Nineteenth-Century Britain*, Oxford: Oxford University Press, 2011, pp. 163–195.

3 Pierre Bourdieu, *Homo Academicus* (tr. Peter Collier), Cambridge: Polity, 1988; Rina Knoeff and Robert Zwijnenberg (eds.), *The Fate of Anatomical Collections*, Farnham: Ashgate, 2015; Steven Lubar, Lukas Rieppel, Ann Daly and Kathrinne Duffy, ‘Lost museums’, *Museum History Journal* (2017) 10, pp. 1–14; Boris Jardine, Emma Kowal and Jenny Bangham, ‘How collections end: objects, meaning and loss in laboratories and museums’, this issue.

4 Hildebrandt, op. cit. (2).

5 Flemming to Kurator, 3 May 1902, Geheimes Staatsarchiv Preußischer Kulturbesitz (hereafter GStA PK), I. HA Rep. 76 Kultusministerium, V^a^ Sekt. 9 Tit. IV Nr. 3 Bd. 4, Bl. 6; F. Graf v. Spee, ‘Walther Flemming†’, *Anatomischer Anzeiger* (1906) 28, pp. 41–59.

6 Hartwin Spenkuch, ‘Einleitung: Republikanische Wissenschaftspolitik im Freistaat Preußen: Problemlagen, Professorenberufungen, Leistungen’, in BBAW and Wolfgang Neugebauer (eds.), *Acta Borussica, Neue Folge*, ser. 2: *Preußen als Kulturstaat*, part II: *Der preußische Kulturstaat in der politischen und sozialen Wirklichkeit*, vol. 9: *Wissenschaftspolitik in der Weimarer Republik: Dokumente zur Hochschulentwicklung im Freistaat Preußen und zu ausgewählten Professorenberufungen in sechs Disziplinen (1918 bis 1933)*, Berlin: de Gruyter, 2016, pp. 1–186, 444–464, 37–38.

7 For the retirement see Kurator to Minister für Wissenschaft, Kunst und Volksbildung, 11 October 1922 and 19 April 1923, and Minister to Spee and to Kurator (office copies), 11 May 1923, GStA PK, I. HA Rep. 76 Kultusministerium, V^a^ Sekt. 9 Tit. IV Nr. 3 Bd. 6, Bl. 369, 389–390.

8 On Benninghoff see Marielene Putscher, ‘Alfred Benninghoff (1890–1953)/Anatom’, in Ingeborg Schnack (ed.), *Marburger Gelehrte in der ersten Hälfte des 20. Jahrhunderts*, Marburg: Elwert, 1977, pp. 31–36; Loremarie Rauch, ‘Alfred Benninghoff 1890–1953’, diss. med. dent., Heinrich-Heine-Universität Düsseldorf, 1990; and Kornelia Grundmann and Gerhard Aumüller, ‘Anatomen in der NS-Zeit: Parteigenossen oder Karteigenossen? Das Marburger Anatomische Institut im Dritten Reich’, *Medizinhistorisches Journal* (1996) 31, pp. 322–357, 345–354; for his appointments see Landesarchiv Schleswig-Holstein (hereafter LASH), Abt. 47.6 Nr. 54, Bl. 1–15; and Archiv der Philipps-Universität Marburg im Hessischen Staatsarchiv Marburg (hereafter UniA MR), Best. 310 Nr. 2327.

9 Spee contributed to a bronze relief of Flemming for the institute: Spee to Kurator, 12 June 1908 (copy), GStA PK, I. HA Rep. 76 Kultusministerium, V^a^ Sekt. 9 Tit. X Nr. 3 Bd. 2, Bl. 21. Flemming’s ‘bust’ later shared the foyer with portraits of Spee, Benninghoff and others: ‘Mit Mikroskop, Seziermesser und Kamera …: NR-Besuch im Anatomischen Institut der Christian-Albrechts-Universität Kiel’, *Nordische Rundschau*, 20 January 1942, clipping in Universitätsbibliothek Marburg, Depositum Benninghoff (hereafter DB), Box 10, folder ‘Abgeschlossene Akten’.

10 Heinrich Schmidt, *Ernst Haeckel und sein Nachfolger Professor Dr Ludwig Plate*, Jena: Volksbuchhandlung, 1921.

11 A. Dabelow, ‘Alfred Benninghoff†’, *Deutsche medizinische Wochenschrift* (1953) 78, pp. 788–789 (quotations); Rauch, op. cit. (8), p. 7 (socializing). On Möllendorff see W. Bargmann, ‘Wilhelm v. Möllendorff† (6.12.1887–10.2.1944)’, *Zeitschrift für Zellforschung und mikroskopische Anatomie, Abt. A* (1945) 33, pp. 167–186, 168, 178–179; Gian Töndury, ‘Wilhelm von Möllendorff†, geb. 6.12.1887, gest. 10.2.1944’, *Vierteljahrsschrift der Naturforschenden Gesellschaft in Zürich* (1944) 89, pp. 141–145, 141–142; Benno Maria Zurgilgen, *Der Anatom Wilhelm von Möllendorff (1887–1944)*, Zurich: Juris, 1991, p. 50.

12 A. Benninghoff, ‘Abschiedsrede vom 12.XII 1940’, fol. 4 (quotation), DB, Box 10, folder ‘Vorträge – Reden’; Rauch, op. cit. (8), p. 57; for Anne-Marie Benninghoff as ‘my most faithful collaborator’ see Alfred Benninghoff, *Lehrbuch der Anatomie des Menschen: Dargestellt unter Bevorzugung funktioneller Zusammenhänge*, 2 vols. in 3, Munich: Lehmann, 1939–1942, vol. 2, part 1: *Eingeweide*, p. vi.

13 A. Benninghoff, ‘Ernst Göppert†’, *Anatomischer Anzeiger* (1952) 98, pp. 410–420, 420, recalled how, from an ‘out-of-the-way’ room, Göppert ‘reigned quietly and discreetly like a good spirit over his old [Marburg] institute’, never interfering; ‘he always kept a certain distance … which was happily combined with a warm, human concern’.

14 On aristocratic science see Mary Jo Nye, ‘Aristocratic culture and the pursuit of science: the de Broglies in modern France’, *Isis* (1997) 88, pp. 397–421; and on the Weimar professoriate, for example, Herbert Döring, *Der Weimarer Kreis: Studien zum politischen Bewußtsein verfassungstreuer Hochschullehrer in der Weimarer Republik*, Meisenheim am Glan: Hain, 1975. Before Spee’s promotion in 1902, his wife, Countess Anna, used an audience with Wilhelm II, on his yacht *Hohenzollern*, to require the Kultusminister to keep His Majesty informed; but Spee, a local who had deputized successfully, faced no serious competition: GStA PK, I. HA Rep. 76 Kultusministerium, V^a^ Sekt. 9 Tit. IV Nr. 3 Bd. 4, Bl. 37–39, 51–59.

15 For those present see Spee to Wilhelm Graf von Spee, 11 April 1925, Archiv der Grafen von Spee, Schloss Heltorf, Düsseldorf, T 27; for Keibel’s role see Benninghoff, op. cit. (1), p. 337; and for Möllendorff’s see preface to *Zeitschrift für Anatomie und Entwicklungsgeschichte* (1925) 76.

16 A draft called the coat ‘shabby’: A. Benninghoff, ‘Erinnerungen an den Anatomen Graf F. v. Spee’, fol. 1, DB, Box 8, thick cardboard wrapper.

17 Benninghoff, ‘Abschiedsrede’, op. cit. (12).

18 Wilhelm Anschütz to Anne-Marie Benninghoff, 20 February 1953, DB, Box 6, folder ‘Teilnehmer an der Trauerfeier, Liste Nr. 4’.

19 *Anatomischer Anzeiger* (1935) 80, p. 160; ‘Trauerfeier für Geheimrat Prof. Dr Ferdinand Graf von Spee’, *Kieler Neueste Nachrichten*, 18 March 1937, clipping in LASH, Abt. 47 Nr. 1595; Benninghoff, ‘Abschiedsrede’, op. cit. (12) (quotations).

20 Benninghoff, ‘Abschiedsrede’, op. cit. (12), all emphases original.

21 On slides see Ilana Löwy (ed.), *Microscope Slides: Reassessing a Neglected Historical Resource*, special issue, *History and Philosophy of the Life Sciences* (2013) 35(3).

22 Wilhelm v. Möllendorff, ‘Ferdinand Graf v. Spee zum 70. Geburtstag’, *Deutsche medizinische Wochenschrift* (1925) 51, p. 573.

23 For the method see F. Graf Spee, ‘Beitrag zur Entwicklungsgeschichte der frühen Stadien des Meerschweinchens bis zur Vollendung der Keimblase’, *Archiv für Anatomie und Entwickelungsgeschichte* (1883), pp. 45–60 and Plate II, 47–49; on Hensen the embryologist see Rüdiger Porep, *Der Physiologe und Planktonforscher Victor Hensen (1835–1924): Sein Leben und sein Werk*, Neumünster: Wachholtz, 1970, pp. 81–84; and Nick Hopwood, *Haeckel’s Embryos: Images, Evolution, and Fraud*, Chicago: The University of Chicago Press, 2015, pp. 218–220.

24 Spee’s published account of the preparation of embryo v.H.: F. Graf v. Spee, ‘Neue Beobachtungen über sehr frühe Entwickelungsstufen des menschlichen Eies’, *Archiv für Anatomie und Entwickelungsgeschichte* (1896), pp. 1–30 and Plate I, 4–5. Further: Jochen Krüger, ‘Die Implantation des Keimes in die Uteruswand: Eine historische Betrachtung unter besonderer Berücksichtigung des Kieler Anatomen Ferdinand Graf von Spee’, diss. med., Christian-Albrechts-Universität zu Kiel, 1969; Nick Hopwood, ‘Producing development: the anatomy of human embryos and the norms of Wilhelm His’, *Bulletin of the History of Medicine* (2000) 74, pp. 29–79; Hopwood, *Embryos in Wax: Models from the Ziegler Studio*, Cambridge: Whipple Museum of the History of Science, 2002.

25 Founded in 1914, the collection of the Carnegie Institution of Washington Department of Embryology incorporated some foreign specimens. The standard survey mentions Spee’s and notes their destruction in the Second World War: Ronan O’Rahilly and Fabiola Müller, *Developmental Stages in Human Embryos, Including a Revision of Streeter’s ‘Horizons’ and a Survey of the Carnegie Collection*, Washington, DC: Carnegie Institution of Washington, 1987, pp. 51 (‘unsatisfactory’), 79, 104 (‘destroyed’), 121, 138. Further on the Carnegie Department see Lynn M. Morgan, *Icons of Life: A Cultural History of Human Embryos*, Berkeley: University of California Press, 2009.

26 Alfred Benninghoff, ‘Über den funktionellen Bau des Knorpels’, *Verhandlungen der Anatomischen Gesellschaft* (1922) 31, pp. 250–267; Benninghoff, ‘Die Anatomie funktioneller Systeme’, *Gegenbaurs Morphologisches Jahrbuch* (1930) 65, pp. 1–10; Benninghoff, *Lehrbuch*, op. cit. (12); Detlev Drenckhahn and Jens Waschke (eds.), *Benninghoff Taschenbuch Anatomie*, 2nd edn, Munich: Urban & Fischer/Elsevier, 2014. For sustained engagement with Roux see Alfred Benninghoff, ‘Form und Funktion’, *Zeitschrift für die gesamte Naturwissenschaft* (1935) 1, pp. 149–160, (1936) 2, pp. 102–114; and Benninghoff, ‘Eröffnungsvortrag’, *Verhandlungen der Anatomischen Gesellschaft* (1938) 46, pp. 6–22, esp. 13–14; on Benninghoff’s unacknowledged debt to Braus see Robert Herrlinger, ‘Wandlungen im anatomischen Unterricht seit Hermann Braus’, *Sudhoffs Archiv* (1953) 37, pp. 266–277, esp. 275.

27 Following Möllendorff, op. cit. (22). Spee in fact habilitated in ‘anatomy and embryology’: Dean Arnold Heller to Kurator, 29 April 1885, GStA PK, I. HA Rep. 76 Kultusministerium, V^a^ Nr. 10207, Bl. 36. In 1898, already an associate professor, Spee was appointed to a new ‘extraordinary professorship of anatomy’: ibid., Sekt. 9 Tit. IV Nr. 3 Bd. 3, Bl. 199–200, 207–212.

28 Transplantation of teeth was out of vogue, but the many other transplants in the period perhaps lent tooth-bud transplantation plausibility: Henry W. Noble, ‘Tooth transplantation: a controversial story’, *Newsletter (History of Dentistry Research Group)* (October 2002) 11, at www.historyofdentistry.group/index_htm_files/2002oct4.pdf, accessed 4 October 2018; Thomas Schlich, *The Origins of Organ Transplantation: Surgery and Laboratory Science, 1880–1930*, Rochester, NY: University of Rochester Press, 2010.

29 Graf F. Spee, ‘Zur Vorweisung von Präparaten menschlicher Lungen, die in natürlicher Spannung konserviert wurden’, *Verhandlungen der Anatomischen Gesellschaft* (1928) 37, pp. 302–306. On frozen sections see Salim Al-Gailani, ‘The “ice age” of anatomy and obstetrics: hand and eye in the promotion of frozen sections around 1900’, *Bulletin of the History of Medicine* (2016) 90, pp. 611–642.

30 For a review that distributes credit a little more see Alfred Benninghoff, ‘Anatomische Beiträge zur Frage der Verschiebung der Ventilebene im Herzen’, *Ärztliche Forschung* (1948) 2, pp. 27–32.

31 Benninghoff, ‘Eröffnungsvortrag’, op. cit. (26), p. 18. For a prospectus of, and clip from, Benninghoff and K. Nitzschke, ‘Über den Gestaltwechsel des Herzens während seiner Tätigkeit’ (1936), see http://vlp.mpiwg-berlin.mpg.de/library/data/lit38405, accessed 5 January 2018; further see Ulf Schmidt, *Medical Films, Ethics and Euthanasia in Nazi Germany: The History of Medical Research and Teaching Films of the Reich Office for Educational Films/Reich Institute for Films in Science and Education*, *1933–1945*, Husum: Matthiesen, 2002.

32 Benninghoff again followed Möllendorff, op. cit. (22).

33 Benninghoff, ‘Abschiedsrede’, op. cit. (12), fols. 4–5.

34 The concert experience was standardized by this time: Hansjakob Ziemer, *Die Moderne hören: Das Konzert als urbanes Forum*, 1890–1940, Frankfurt am Main: Campus, 2008.

35 According to H. Feneis, ‘Klaus Niessing†’, *Anatomischer Anzeiger* (1965) 116, pp. 417–422, 419, Niessing ‘greatly admired’ Spee. Altered passages in Benninghoff’s manuscript suggest that respect coexisted with occasional ridicule (see below).

36 *Kiel als Universitätsstadt*, Kiel: Mühlau, 1921, p. 22. For numbers of medical students see Hanns Löhr, ‘Die medizinische Fakultät’, in Paul Ritterbusch, Hanns Löhr, Otto Scheel and Gottfried Ernst Hoffmann (eds.), *Festschrift zum 275jährigen Bestehen der Christian-Albrechts-Universität Kiel*, Leipzig: Hirzel, 1940, pp. 164–215, 209.

37 Möllendorff, op. cit. (22).

38 Kurator to Minister, 12 June 1924, GStA PK, I. HA Rep. 76 Kultusministerium, V^a^ Sekt. 9 Tit. X Nr. 3 Bd. 2, Bl. 256–257.

39 For example, Carl Rabl, *Geschichte der Anatomie an der Universität Leipzig*, Leipzig: Barth, 1909, pp. 107–118.

40 Möllendorff to Kurator, 17 May 1925, GStA PK, I. HA Rep. 76 Kultusministerium, V^a^ Sekt. 9 Tit. X Nr. 3 Bd. 2, Bl. 284–287. On Ziegler models see Hopwood, *Embryos in Wax*, op. cit. (24).

41 Möllendorff to Kurator, op. cit. (40). On renewing and handling see Hieke Martine Huistra, ‘Preparations on the move: the Leiden anatomical collections in the nineteenth century’, doctoral dissertation, Universiteit Leiden, 2013, pp. 11–41; for the introduction of learning collections see W. Tonkoff, ‘Über die Einrichtung der anatomischen Lernsammlungen’, *Anatomischer Anzeiger* (1906) 29, pp. 479–489.

42 Kurt Feyerabend, *Die Universität Kiel: Ihre Anstalten, Institute und Kliniken*, Düsseldorf: Lindner, [1929], p. 34.

43 Benninghoff, op. cit. (16), fol. 12.

44 Benninghoff, op. cit. (16), fol. 12.

45 Grundmann and Aumüller, op. cit. (8), pp. 345–354; Sabine Hildebrandt, ‘Anatomische Gesellschaft from 1933 to 1950: a professional society under political strain – the Benninghoff papers’, *Annals of Anatomy* (2013) 195, pp. 381–392; Karl-Werner Ratschko, *Kieler Hochschulmediziner in der Zeit des Nationalsozialismus: Die Medizinische Fakultät der Christian-Albrechts-Universität im ‘Dritten Reich’*, Essen: Klartext, 2014, pp. 45–46, 362–364, 501–503; more generally see Hildebrandt, op. cit. (2); and Andreas Winkelmann, ‘The Anatomische Gesellschaft and National Socialism: an analysis based on newly available archival material’, *Annals of Anatomy* (2015) 201, pp. 17–30.

46 Ratschko, op. cit. (45); for the quotation see Benninghoff to Spruchkammer I, Marburg-Stadt, 24 October 1946, DB, Box 10, folder ‘Abgeschlossene Akten’.

47 A. Benninghoff, ‘Die Wandlung der geistigen Grundlagen in Biologie und Medizin. Fachschaftsvortrag von Professor Benninghoff am 13.VI.34’, fols. 6–7, DB, Box 10, folder ‘Vorträge – Reden’; see also Benninghoff, ‘Ansprache an die Mediziner. Beginn W.S. 1937/38’, ibid. Pace Olivier Rieppel, ‘Karl Beurlen (1901–1985), nature mysticism, and Aryan paleontology’, *Journal of the History of Biology* (2011) 45, pp. 253–299, 256, 281–282, Benninghoff’s brief founding co-editorship of the *Zeitschrift für die gesamte Naturwissenschaft*, when based in Kiel and before its radicalization, indicates that he worked with ideological Nazis, but not that he endorsed their ‘Aryan science’.

48 A. Benninghoff, ‘Medizinervollversammlung 13.4.38’, fols. 2–3, DB, Box 10, folder ‘Vorträge – Reden’.

49 A. Benninghoff, ‘FestE[ssen]’, DB, Box 10, folder ‘Vorträge – Reden’.

50 Benninghoff to Spruchkammer I, Marburg-Stadt, 24 October 1946 (quotation) and to Spruchkammer Marburg Stadt, [autumn 1947], DB, Box 10, folder ‘Abgeschlossene Akten’.

51 Ratschko, op. cit. (45), pp. 502–503.

52 On Austria see Benninghoff, op. cit. (48), fol. 1; and A. Benninghoff, ‘Ansprache’, *Verhandlungen der Anatomischen Gesellschaft* (1938) 46, pp. 3–4.

53 Quoted in Grundmann and Aumüller, op. cit. (8), p. 348.

54 For the leave see Benninghoff to Dean, 24 August 1942, UniA MR, Best. 307c Nr. 5105; and to Reichsminister, 29 October 1943, ibid., Best. 305a Nr. 4007, Bl. 56.

55 Griese to Benninghoff, 27 November 1943, DB, Box 10, folder ‘Abgeschlossene Akten’.

56 Benninghoff to Spruchkammer Marburg Stadt, [autumn 1947], DB, Box 10, folder ‘Abgeschlossene Akten’.

57 Benninghoff, op. cit. (56). Before his Marburg appointment, the NSDAP judged that Benninghoff had ‘done nothing that was in any way directed against the Nat[ional]-Soc[ialist] state’: Gaudozentenbundesführer Bersin to Rector, 21 October 1940, UniA MR, Best. 305a Nr. 4007, Bl. 9. In general see Herbert Mehrtens, ‘Irresponsible purity: the political and moral structure of mathematical sciences in the National Socialist state’, in Monika Renneberg and Mark Walker (eds.), *Science, Technology and National Socialism*, Cambridge: Cambridge University Press, 1994, pp. 324–338, 411–413; on anatomy see Hildebrandt, op. cit. (2), pp. 258–295; and Winkelmann, op. cit. (45).

58 A. Benninghoff, ‘Immatrikulationsfeier, 28.11.1951’, fol. 4, DB, Box 1, folder ‘Anatomen-Gesellschaft’.

59 Hildebrandt, op. cit. (2). Some anatomists did worse; see also Paul Weindling, *Victims and Survivors of Nazi Human Experiments: Science and Suffering in the Holocaust*, London: Bloomsbury Academic, 2015, pp. 119–125.

60 Hildebrandt, op. cit. (2), p. 275.

61 On the bombing see Karl-Werner Ratschko, ‘Kieler Hochschulmedizin im Krieg: Zerstörung der Kliniken und medizinischen Institute im 2.Weltkrieg’, *Schleswig-Holsteinisches Ärzteblatt* (2009) 62(1), pp. 56–61, 60; on the post-war ruin see Wolf Herre, ‘Die Christian-Albrechts-Universität zu Kiel nach 1945’, in Brigitte Schauenburg (ed.), *750 Jahre Kiel: Beiträge zu Geschichte und Gegenwart der Stadt*, Kiel: Mühlau, 1992, pp. 141–156, 142, 147–149, 152.

62 Julia Buddecke, *Endstation Anatomie: Die Opfer nationalsozialistischer Vernichtungsjustiz in Schleswig-Holstein*, Hildesheim: Olms, 2010.

63 Göppert to Benninghoff, 6 December 1936, DB, Box 10, folder ‘Abgeschlossene Akten’.

64 Benninghoff to Johannes Hett (copy), 21 August 1950, DB, Box 9, folder ‘20. Mai 1949 bis 29. Dez. 1950’: Flemming’s widow had sold his slides to Lund. ‘During his life as emeritus Count Spee kept all his material including the microsc[opical] demonstration collection for himself. V[on] Möllendorf[f] took all his material with him from Kiel to Freiburg.’

65 Rauch, op. cit. (8), p. 67; also Putscher, op. cit. (8), p. 33. I have yet to find the ‘many letters’ that Rauch reported in Benninghoff’s (uncatalogued) papers.

66 Rostock to Benninghoff, 28 December 1944 (office copy), Bundesarchiv, R 9361/62293, Bl. 930.

67 Schröder to Benninghoff, 31 October 1944, DB, Box 7, beige folder.

68 Quotation: Benninghoff, untitled farewell speech to friends and colleagues at home, December 1940, DB, Box 1, brown A5 envelope.

69 Netter to Benninghoff, 21 November [1945], and Benninghoff to Netter, 30 November 1945 (copy), DB, Box 1, brown A5 envelope; Netter to Benninghoff, 22 September 1949 and Benninghoff to Netter, 26 September 1949 (copy), DB, Box 9, folder ‘20. Mai 1949 bis 29. Dez. 1950’.

70 Ratschko, op. cit. (45), pp. 496–498.

71 Two ring binders in the Institut für Anatomie und Embryologie, Zentrum Anatomie, Universität Göttingen, document the story; see especially Erich Blechschmidt to President Norbert Kamp, 17 July 1988: ‘This request corresponds to an old tradition, that research collections remain for the length of his life in the possession of the emeritus who built them up for his research and from whom scientific work is expected even after his retirement.’ On Blechschmidt, see the in some respects problematic Florian G. Mildenberger, ‘Anatom, Abtreibungsgegner, Antidarwinist: Die drei Leben des Erich Blechschmidt (1904–1992)’, *Medizinhistorisches Journal* (2016) 51, pp. 246–279.

72 An American reflection, also from 1944, Harris P. Mosher, ‘On being a professor emeritus’, reprinted in *Annals of Otology, Rhinology & Laryngology* (1994) 103, pp. 919–928, includes the rules ‘Give advice only when asked. Don’t expect to be asked … Don’t feel openly hurt if your pet instruments, especially those you invented, are not used, but are put in the historical collection.’

